# Primary low grade myxofibrosarcoma of the liver with benign presentation but malignant outcome: a case report

**DOI:** 10.1186/s12885-019-6282-0

**Published:** 2019-11-12

**Authors:** Zigong Shao, Baoping Jiao, Juanhan Yu, Hao Liu

**Affiliations:** 1 Department of General Surgery, Department of Organ Transplantation, First Affiliated Hospital, China Medical University, Shenyang, 110001 China; 2 Department of General Surgery, Shanxi Cancer Hospital, Taiyuan, 130013 China; 3 Department of Pathology, First Affiliated Hospital, China Medical University, Shenyang, 110001 China

**Keywords:** Myxofibrosarcoma, Liver neoplasms

## Abstract

**Background:**

Myxofibrosarcoma (MFS) is most often found on the limbs of aged male people, but extremely uncommon in the liver.

**Case presentation:**

A 52-year-old female patient with a liver mass was diagnosed as a primary MFS. It had no obvious abdominal symptoms, and the tumor was resected with an extended margin. Three years after the surgery, the patient was readmitted for peritoneal metastasis and passed away 4 months later. The tumor has a benign presentation, but malignant outcome.

**Conclusions:**

Comprehensive radiological inspection, intensive preoperative evaluation, careful design of operating procedures, wide margin resection, consecutive treatment, and strict periodical follow-ups should be taken to ensure a better prognosis of this kind of neoplastic disease.

## Background

Angervall et al. first described myxofibrosarcoma (MFS) in 1977 as a class of fibroblastic neoplasm, which showed a wide range of cellularity, nuclear polymorphism and proliferative capability [1]. Weiss [2] and Hollowood [3] then classified MFS into low (myxoid mainly), intermediate (mixed cell and myxoid), and high (predominantly cellular) grade types.

MFS is most often found on the limbs of aged male people, but extremely uncommon in the liver. Rare occurrences have been reported in cranial cavity [4], orbit [5], maxilla [6], parotid gland [7], hypopharynx [8], sinus piriformis [9], vocal folds [10], thyroid gland [11], esophagus [12], breast [13], heart [14], aorta [15], scapular region [16], buttock [17], and scrotum [18]. By literature review, hepatic MFS has never been reported previously except one metastasis [19]. We here report the first case of primary low grade MFS of the liver.

## Case presentation

A 52-year-old female patient with no obvious abdominal symptoms was admitted for liver mass found by ultrasound examination. A hypoechoic solid mass ranged 5.7 * 5.6 * 5.0 cm was detected in the left lobe, with a clear outline and abundant blood flow inside the tumor. Physical examination showed no jaundice in the skin and sclera, liver palms, and any spider angioma. The abdomen was flat and soft, with no varicose veins. No tenderness or rebound pain was induced by palpation. All investigations of chest and limbs were normal. Laboratory tests showed normal blood cell count and liver functions except a slightly increase of total bilirubin to 21.3 μmol/L. Liver tumor related cell markers, including alpha feto-protein, carcino-embryonic antigen, and carbohydrate antigen 199 were all within normal values. Hepatitis serological tests were all negative. No family or personal history of malignancy disease either.

**Fig. 1:**
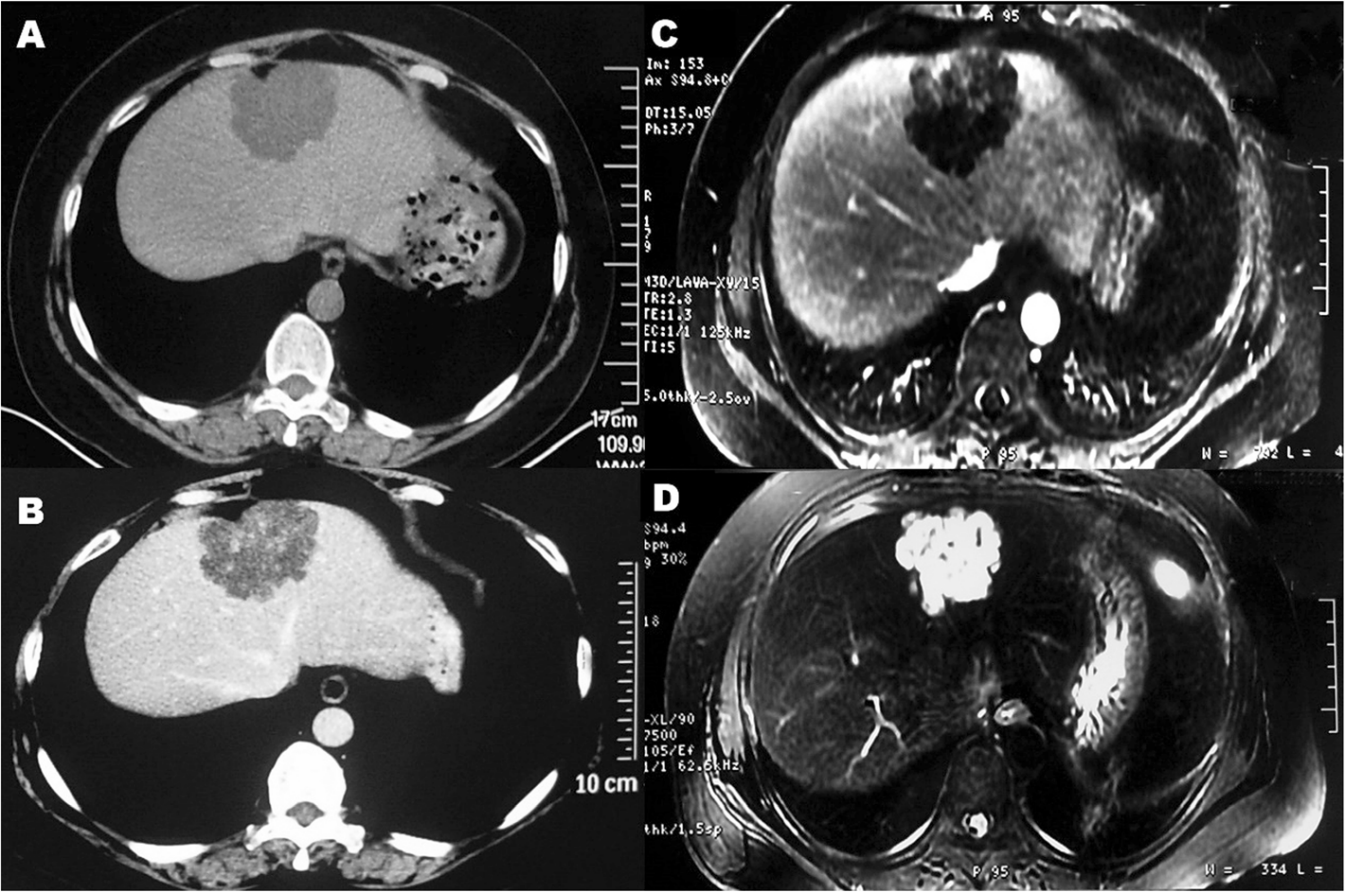
CT and MRI findings of liver mass in IVth segment. **a** plain CT scan. **b** Enhanced CT scan. **c** MRI-T1. **d** MRI-T2

Both CT and MRI examinations revealed the mass in the IVth segment of the liver with a multi-lobular appearance, clear boundary, and internal separations. The solid mass can be enhanced in the CT scan from 20Hu to 30Hu. Long T1 and T2 signal were showed in MRI, with only a weak strengthening in the delayed phase, suggesting the high probability of a benign hepatic tumor (Fig. 1).

**Fig. 2:**
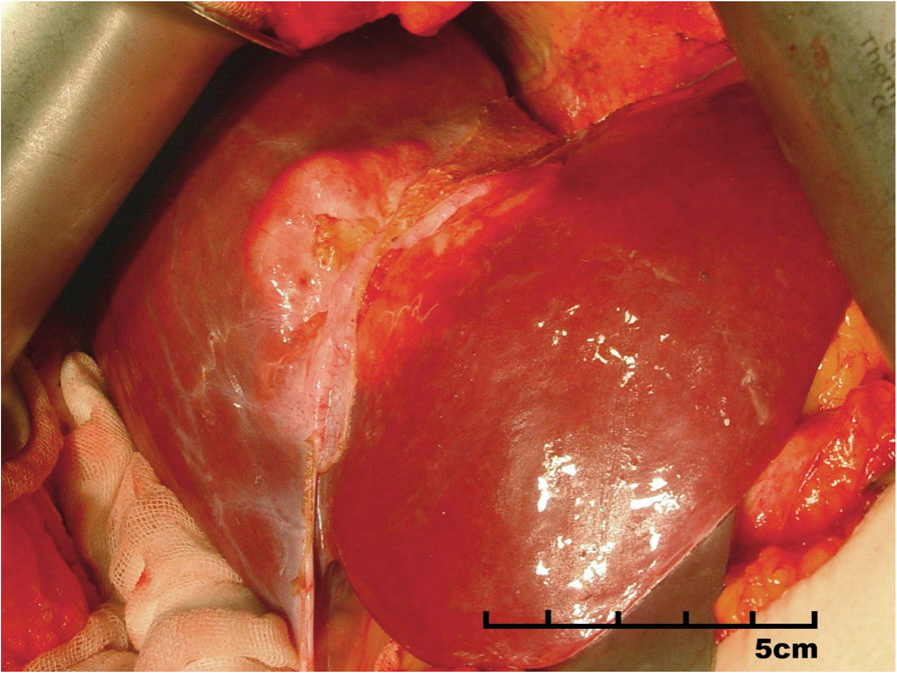
Intraoperative observations. The tumor arises inside the liver with a clear boundary on the surface crossing the falciform ligament

Preoperative biopsy was suggested but denied by the patient and an open abdominal surgery was performed to exclude any risk of malignancy. The tumor was identified on the diaphragm side of the liver, with a size of 6 * 5 * 5 cm and white-yellow color, in an exophytic growth pattern (Fig. 2).

**Fig. 3:**
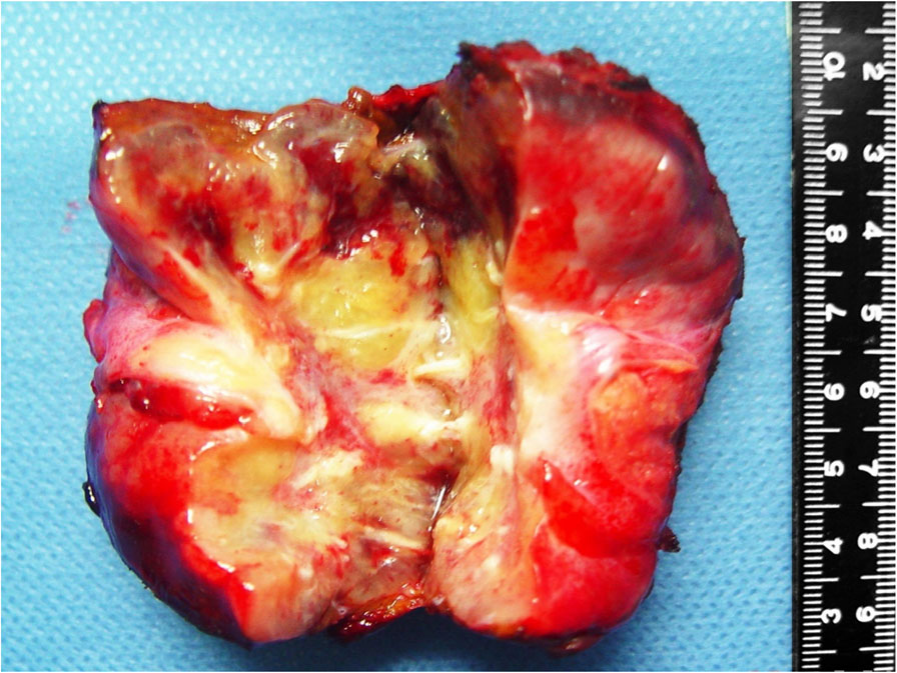
Macroscopic view of the resected liver tumor

The tumor was resected, and no intraoperative frozen sections were taken because the mass was recognized as a benign lesion. A smooth capsule with a clear boundary can be visualized outside the solid-cystic cortex, containing yellow colored jelly-like substance in the central portion with white fibrous septa (Fig. 3).

**Fig. 4:**
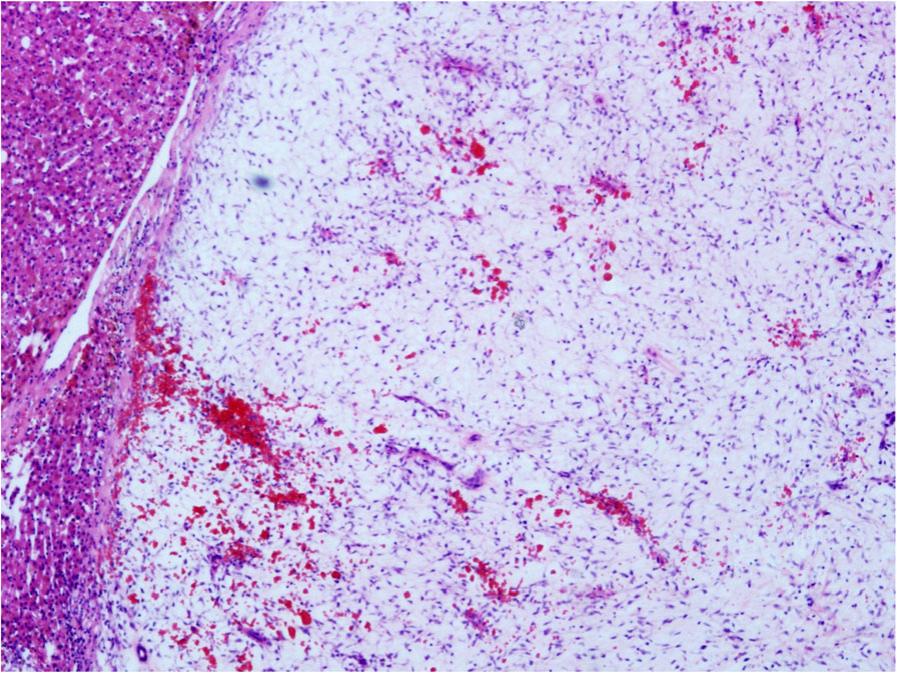
Microscopic view of resected liver mass, HE staining × 4

Microscopy findings showed that the tumor was composed with different proportions of loose fibroelastic connective tissue, hypocellular mucus-containing stroma, and scattered bile duct epithelium, associated with partial hemorrhage (Fig. 4).

**Fig. 5:**
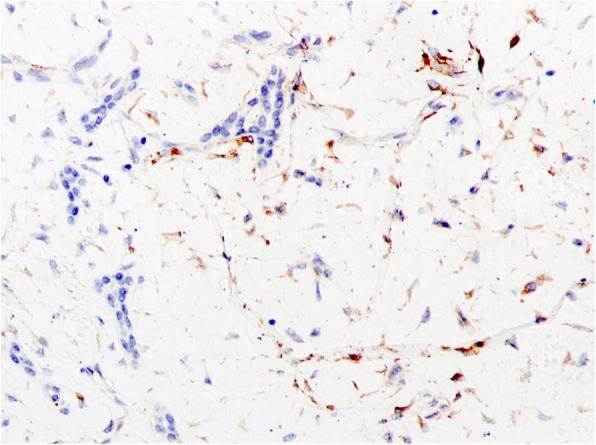
Immunohistochemistry staining of vimentin × 20

Immunohistochemistry (IHC) showed staining of Vimentin (Fig. 5), SMA, CK, CK7, CD34, < 2% Ki67, but no CD117 and S^− 100^. Pathological diagnosis was collectively determined as the hepatic mesenchymal hamartoma by three pathological experts. According to AJCC classification, this mass belongs to T2bN0M0/IIb. Based on FNCLCC system, the hepatic tumor belongs to Grade 1 and the nodules of omentum metastasis belongs to Grade 2.

**Fig. 6:**
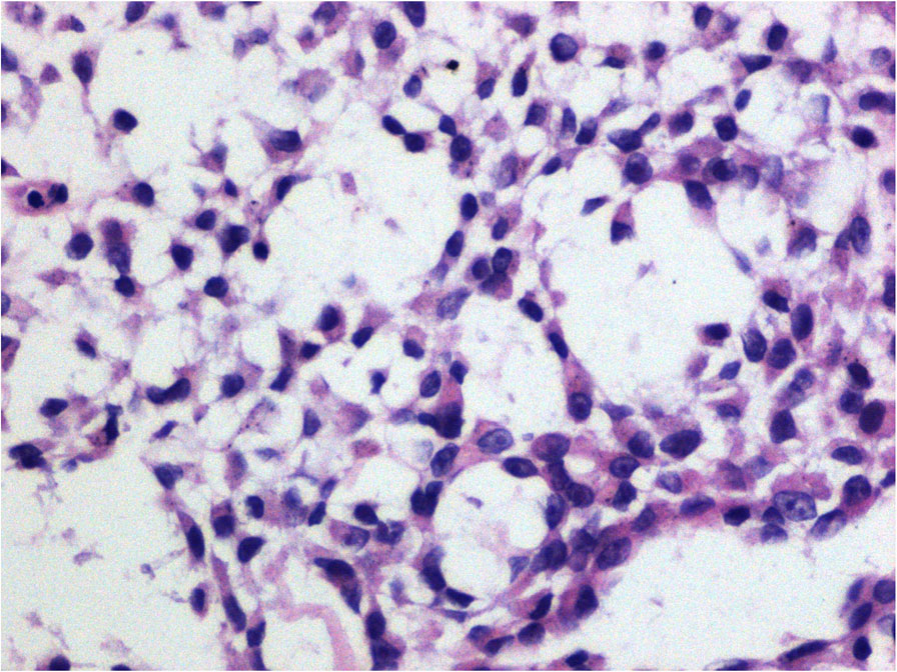
H.E. staining of abdominal biopsy specimen × 40

Three years after the surgery, the patient was readmitted because of massive ascites. Severe peritoneal effusions and omental thickening were detected by both ultrasound and enhanced CT scan. An ultrasound-guided percutaneous biopsy of the omentum was performed and followed by H.E. staining. It was found that spindle-shaped cells were distributed in a bundled or interlaced pattern with a relative high density. Multi-sized nuclei were slightly enlarged and eosinohilic cytoplasms were transparent but lack clear boundaries with surrounding myxoid stroma (Fig. 6).

IHC staining of the biopsy specimen showed positive of vimentin, calretinin, and D2–40, < 5% Ki67, but weak for CK and negative for CK7, CK20, SMA, CEA, CA125, GLUT-1, WT1, CD34, and S^− 100^. Combination of the clinical progress with histopathological findings indicated that these metastatic lesions were developed from a malignant tumor with a mesenchymal origin, thus considered as low-grade MFS. Pathological consultation was carried out and a historical review of the original liver tumor slices indicated a high homology between these two lesions. The second onset was therefore considered as peritoneal metastasis of the primary liver tumor. This patient passed away 4 months later due to multiple organ failure.

## Discussion and conclusions

Malignant tumors in the liver are predominantly carcinomas, and sarcomas in rare cases. In our report, neither clinical presentation nor radiological examinations revealed the occurrence of a primary tumor elsewhere. This patient had no family or personal history of malignancy either. Unobvious symptoms, non-apparent signs, negative serology tests, near to normal laboratory reports, and untypical radiology appearance misled us to a diagnosis of benign hepatic tumor rather as the MFS of liver.

MFS are classified into two types, solid and “tail-like” pattern by T2-weighted magnetic resonance images (MRI). In “tail-like” type, there are extensive spread along the fascial planes that extended away from the primary site of tumor [20]. MFS with “tail-like” pattern is significantly related to a superficial (subcutaneous) origin, which usually grow in an infiltrative way and have a poor prognosis [21, 22]. However, it should be noted that the solid type appearance of MFS does not necessarily mean negative metastases, as it happened in our patient.

If a biopsy was performed to suggest a diagnosis of MFS, a thorough radiological investigation to estimate the tumor extension should be mandatory. Due to its infiltrative nature, a much wider margin should be applied even for a low or intermediate-grade MFS to prevent a local recurrence [23]. In case of incomplete resection, extra revision surgery should be systematically performed [24]. Unfortunately, initial biopsy was denied in our case and although we removed the tumor with an extended margin, it may be not really adequate as demanded in MFS treatment. In addition, initial pathological findings of the resected liver tumor were not distinct enough to diagnose MFS after the surgery.

In general, low grade MFS is shown as a jelly-like multinodular tumor with a weakly invasive pattern of growth. Under microscope, spindle or stellate-shaped tumor cells are mainly distributed on a mucoid matrix (50%), mainly consists of hyaluronic acid [25]. Extra features include curvilinear elongated blood vessels and occasional existence of pseudolipoblasts. However, no specific findings were identified in our patient, and the existence of bile duct epithelium (CK and CK7 staining in IHC) misled the pathological diagnosis to the benign hamartoma. In fact, it would be very difficult to make the primary diagnosis of liver MFS even with a preoperative biopsy. It was not until the development of extensive peritoneal metastases leading to percutaneous biopsy that MFS was suggested. A thorough microscopic comparison of two pathological specimens Similarities include the presence of plump spindled tumor cells with hyperchromatic nuclei, predominant mucous stroma, and IHC staining of vimentin. However, CK and CK7 staining in the peritoneal specimen were quite different from those in hepatic tumor, may be due to the presence of adjacent liver tissue.

Stringent follow-up was not required in our patient because there was no report of metastasis in hamartoma. In contrast, although low grade MFS is considered to have less potential of malignancy, it shows considerable frequency of metastases (about 20 to 25%) [1, 20]. Metastases of MFS are most common in brain, lung, stomach, small bowel, adrenal gland, pelvis, and retroperitoneum [26]. Larger size (over 5 cm) and necrosis are the most predictors of metastasis [25]. Tumor size, pathological grade, and surgical margins were statistically significant predictors of survival [27]. Primary operation of MFS without careful evaluation and specific plan is the most significant risk factor associated with poor prognosis [28]. From MRI and CT evaluation before the surgery and also according to the intraoperative observation, there is a clear outline of this intrahepatic mass. Therefore we removed the intact mass with a margin approximately 5 mm from the normal liver tissue. More extended margin was not applied and frozen sections were not taken as it was recognized as a benign tumor. In fact, we agree that the surgical margin might be not adequate, should we know in advance that it developed into a malignant manner. A relative wide margin should be always used if the character of the tumor cannot be precisely determined, as long as the surgical conditions allowed.

To our knowledge, this is the first case report of a primary MFS in the liver. Although the occurrence is remarkably rare, we should learn a lesson and bear in mind of this probability. Comprehensive radiological inspection, intensive preoperative evaluation, careful design of operating procedures, wide margin resection, consecutive treatment, and strict periodical follow-ups should be taken to ensure a better prognosis of this kind of neoplastic disease.

## Data Availability

The data used and/or analyzed during the current study are available from the corresponding author on reasonable request.

## Abbreviations

CT: Computed tomography
HE: Hematoxylin-eosin staining
IHC: Immunohistochemistry
MFS: Myxofibrosarcoma
MRI: Magnetic resonance images
